# Hair Dressing

**Published:** 1889-09

**Authors:** 


### HAIR DRESSING.

It is bad for a woman to soak her hair with water in her daily bath, and yet she may do it with impunity and even with benefit, if she pursues the proper after-treatment. The daily wetting of the hair of the head favors parasitic growths on the hair, and unless the hair is well dried before it is coiled up it will become sour, much to its injury, and give an affensive odor. The hair may be wet daily with impunity, provided after it is wiped as dry as possible it is soaked with alcohol and briskly rubbed with the hands. The alcohol stops the parasitic growth, and in evaporating carries off the last of the water, thus preventing all tendancy to sourness and enabling the hair to be put up promptly.

The disease of the scalp which produces dandruff eventually produce baldness and frequently quickly. Persons who want to preserve their fine heads of hair have to give them the same good attention they give other valuable possessions. If there is much dandruff the head should have a thorough dressing twice a week. A dressing should commence with the careful use of a small tooth-comb until all dandruff in sight is removed. This should be followed with a thorough washing with warm water and white castile soap. Every portion of the scalp should be well rubbed and afterwards every trace of the soap should be washed away with moderately warm water.

